# Genome Sequence of *Paraburkholderia nodosa* Strain CNPSo 1341, a N_2_-Fixing Symbiont of the Promiscuous Legume *Phaseolus vulgaris*

**DOI:** 10.1128/genomeA.01073-16

**Published:** 2016-11-03

**Authors:** Rebeca Fuzinatto Dall’Agnol, Maira Rejane Costa, Renan Augusto Ribeiro, Jakeline Renata Marçon Delamuta, Ligia Maria Oliveira Chueire, Mariangela Hungria

**Affiliations:** a Embrapa Soja, Soil Biotechnology Laboratory, Londrina, Paraná, Brazil; b Department of Biochemistry and Biotechnology, Universidade Estadual de Londrina, Londrina, Paraná, Brazil; c Department of Microbiology, Universidade Estadual de Londrina, Londrina, Paraná, Brazil; d CNPq, SHIS QI 1 Conjunto B, Brasília, Federal District, Brazil; e CAPES, SBN, Quadra 2, Brasília, Federal District, Brazil

## Abstract

*Paraburkholderia nodosa* CNPSo 1341 is a N_2_-fixing symbiont of *Phaseolus vulgaris* isolated from an undisturbed soil of the Brazilian Cerrado. Its draft genome contains 8,614,032 bp and 8,068 coding sequences (CDSs). Nodulation and N_2_-fixation genes were clustered in the genome that also contains several genes of secretion systems and quorum sensing.

## GENOME ANNOUNCEMENT

*Paraburkholderia* has been recently validated as a new genus (1–3) and encompasses several environmental *Burkholderia* (1, 4, 5). The N_2_-fixing *Paraburkholderia* that nodulate legumes are also called beta-rhizobia (6). *Paraburkholderia nodosa* was first isolated from nodules of *Mimosa* (*M. bimucronata* and *M. scabrella*) (7), and later from other leguminous plants from the “*Piptadenia* group” (8), all belonging to the *Mimosoideae* subfamily; more rarely, it nodulates members of the *Papilionoidea* subfamily, as *Phaseolus vulgaris* (9). *P. nodosa* strain CNPSo 1341 was trapped by the promiscuous common bean (*Phaseolus vulgaris*) from an undisturbed soil of the Brazilian Cerrado (9), and here we present its draft genome.

To extract the total bacterial DNA, we used the DNeasy Blood and Tissue Kit (Qiagen) and processed genome sequencing on the MiSeq platform (Illumina) at Embrapa Soja, Londrina, Brazil. Shotgun sequencing generated 2,081,314 paired-end reads (2 × 300 bp), corresponding to approximately a 73.45-fold coverage. The FASTQ files were assembled by the A5-miseq pipeline (*de novo* assembly) (10). The genome of strain CNPSo 1341 was estimated at 8,614,032 bp, with a G+C content of 64.2 mol%, and assembled in 86 contigs, with 8,068 predicted coding sequences (CDSs).

Sequences were submitted to RAST (11) and by analyzing the sequences in the SEED system (11), we determined that 47% of CDSs had coverage in 530 subsystems, the majority in the carbohydrates and amino acids and derivatives categories. The highest genome scores were with *Paraburkholderia* sp. Ch1-1 and *Paraburkholderia xenovorans* LB400, two strains well known for their capacity of degradation of xenobiotics and tolerance of stresses. Indeed, *P. nodosa* CNPSo 1341 carries 249 CDSs related to stress response, 45% of them of oxidative stress, in addition to 204 CDSs of the metabolism of aromatic compounds.

In relation to the symbiosis, the nodulation (*nod* genes) operons were followed by the nitrogen fixation (*nif*) operons. We found one copy of the regulatory *nodD* gene (LysR family) that orchestrates the nodulation process (12), and interestingly, adjacent to the *nodD* there is a gene related to nikkomycin biosynthesis. The genome of *P. nodosa* CNPSo 1341 carries several other CDSs of resistance to and biosynthesis of antibiotics. Preceding the *nifA* there is another regulatory gene of the LysR family that is also present in a plasmid of *Burkholderia* CCGE 1001 and deserves further investigation.

Noteworthy is the variety of secretion systems found in the genome of *P. nodosa* CNPSo 1341. Among them, the complete operon of the secretion system X, which shows greater similarity to the plant pathogen *Ralstonia solanacearum*. In relation to quorum sensing, there is a pair of genes of quorum-sensing LuxR-LuxI, of homoserine lactone, but in addition there are nine other CDSs of transcription regulators of the LuxR family, which might be related to the perception of several signals.

### Accession number(s).

This whole-genome shotgun project has been deposited at DDBJ/EMBL/GenBank under the accession number SUBID SUB1743636 and BioProject PRJNA337952, BioSample SAMN05514346, and accession number MCNV00000000. This paper describes the first version.
